# Low-Dimensional Materials for Future Transistors

**DOI:** 10.1007/s40820-026-02142-7

**Published:** 2026-03-20

**Authors:** Shuohua Zhang, Zhihan Hong, Mingjun Ren, Limin Zhu, Jing Wang

**Affiliations:** School of Mechanical Engineering, Shanghai Jiao Tong University, Shanghai, 200240 People’s Republic of China

**Keywords:** Low-dimensional materials, Transistor scaling, Post-Moore, Lab-to-fab translation, Future transistor

## Abstract

The scaling of transistors is approaching its physical limits, making the future direction of transistor development a topic of global significance. Low-dimensional materials (LDMs), which exhibit superior properties compared to bulk materials, are emerging as a driving force for transformative advancements across various industries. What do LDMs signify for the future of transistors? Where do the challenges and opportunities lie? This perspective concludes with an overview of the transistor development roadmap, highlighting key technological milestones for LDMs, and proposes three pathways for integrating LDMs into future transistors across near-, mid-, and long-term horizons.

**Graphical Abstract:**

**Figure:**
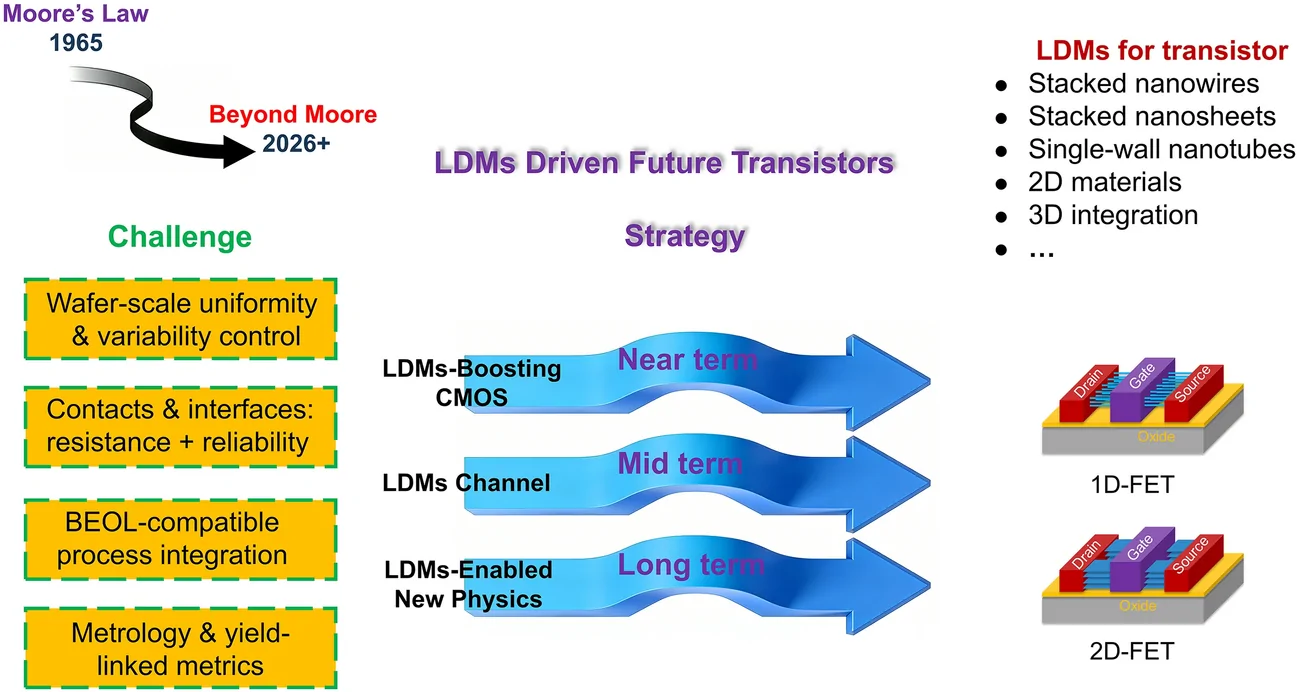

In 1947, the invention of the point-contact transistor, a breakthrough later recognized by the Nobel Prize in Physics, marked the beginning of the microelectronics era (Fig. 1a). Since then, continuous performance improvements through dimensional scaling have transformed human life. In 2025, the Nobel Prize in Physics highlighted macroscopic quantum tunneling, underscoring that quantum effects cannot be ignored in engineered devices. In modern CMOS, analogous quantum effects—direct tunneling across ultrathin dielectrics and source–drain tunneling at nanometer gate lengths—are increasingly becoming first-order constraints on further scaling. As scaling approaches the quantum boundary, must we admit that the next leap in performance demands an entirely new paradigm?

**Fig. 1:**
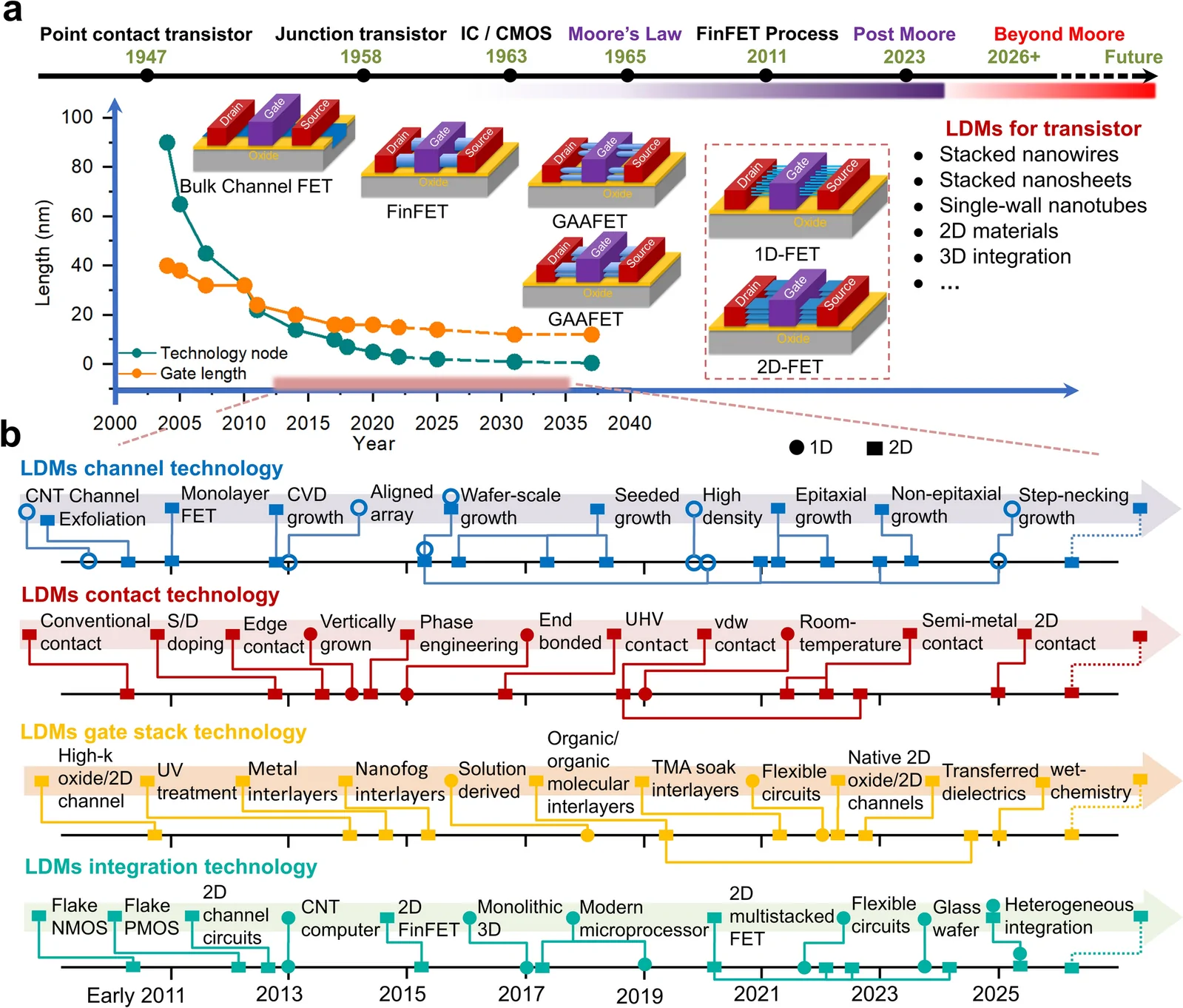
Transistor scaling and the emerging role of low-dimensional materials (LDMs). **a** Evolution of the technology node and physical gate length in CMOS logic. **b** Milestones in LDMs technologies for transistors, including 1D and 2D channel [1, 2, 4]

In 1965, Gordon Moore projected that transistor counts on cost-optimized chips would continue to increase exponentially, often summarized as doubling roughly every 2 years. For decades, the industry largely sustained this trajectory. Since the early twenty-first century, however, continued geometric scaling has been increasingly constrained by power-density limits and exacerbated short-channel effects, weakening electrostatic gate control and diminishing performance gains. In response, architectures such as FinFETs and GAAFETs—along with actively explored CFET (complementary FET) concepts—have strengthened gate–channel coupling and increased effective channel width to suppress short-channel effects. Consequently, progress has relied less on simple dimensional scaling and more on innovations in device architecture and integration under post-Moore scaling. Nevertheless, as conventional channel materials (e.g., Si, strained SiGe, and III–V semiconductors) are thinned toward the few-nanometer regime, quantum confinement and enhanced surface/interface scattering can increase variability and degrade mobility, underscoring the need for new breakthroughs.

Compared to bulk semiconductors, low-dimensional materials (LDMs: at least one dimension is on the nanometer scale) confine carriers in one or more spatial directions, transforming device physics from bulk-like bands into engineered subbands or discrete levels, and pushing transistor operation into a regime dominated by surfaces, interfaces, and electrostatics. Van der Waals materials, with their naturally dangling-bond-free surfaces, offer the potential for high intrinsic mobility. Additionally, their atomic-scale dimensions inherently break through channel size limitations and facilitate 3D stacking. Their high surface-to-volume ratio makes interfaces and contacts first-order design knobs, while atomic thickness enables strong electrostatic control. Figure 1b illustrates the development of LDMs, particularly 2D and 1D materials. As predicted by the International Roadmap for Devices and Systems (IRDS), to sustain the exponential growth of transistor density beyond 2030, monolithic 3D integration—stacking logic devices vertically—is increasingly viewed as a central route [4] (see Fig. 1a). Indeed, in recent years, atomically thin transition metal dichalcogenides (TMDs) and carbon nanotubes have achieved significant advancements in transistor fabrication.

Despite their advantages, low-dimensional materials still face several cross-cutting challenges that ultimately determine circuit-level viability [4]. First, wafer-scale uniformity and defect/variability control of 1D/2D channels remain difficult. Second, contact and interface uncertainties (e.g., contamination, traps, and barrier inhomogeneity) increasingly dominate resistance and reliability as dimensions shrink [2]. Third, process-integration constraints, particularly thermal budget and back-end-of-line (BEOL) compatibility, limit scalable integration options [4]. Finally, standardized metrology/metrics and design–technology co-optimization are required to translate device-level gains into circuit yield and reliability [4]. Accordingly, mitigation strategies prioritize BEOL-compatible integration, wafer-scale process windows, contamination-aware interface/contact modules, and yield-linked metrics, which define priorities from integration feasibility to manufacturability and scalable options [4].

The future development of transistors driven by LDMs can be divided into three branches: LDMs-Boosting CMOS, LDMs Channel, and LDMs-Enabled New Physics (Fig. 2). LDMs-Boosting CMOS integrates LDMs into the mature CMOS architecture, representing a painkiller-like pathway that delivers rapid, engineering-ready gains and includes key research areas such as gate stack & interface engineering, back-end-of-line (BEOL) interconnects, and contact & access resistance reduction. LDMs Channel is the focal point of research and a promising route toward addressing the constraints of Moore’s Law. It encompasses pressing issues like LDM-based FETs, wafer-scale channel fabrication, and 3D integration. The third direction, LDMs-Enabled New Physics, leverages quantum confinement and tunneling in LDMs to explore device concepts beyond classical electrostatics (e.g., tunneling-assisted or steep-slope switching) for future transistors [1, 2]. Although progress may be slow, it promises steady and far-reaching impact, with potential breakthroughs capable of revolutionizing transistor technology. As a representative 1D platform, carbon nanotube (CNT) transistors have advanced from single-tube devices to aligned/array transistors with high current density and near-ballistic transport. However, key bottlenecks remain in semiconductor-purity control, array-level uniformity, and statistically controlled contacts/interconnects [4]. As a representative 2D platform, two-dimensional transistors have progressed from proof-of-concept devices toward system-level integration demonstrations [3], while manufacturability is still constrained by wafer-scale reproducible growth/integration, interface cleanliness, and contact resistance control under BEOL-compatible thermal budgets [2]. Together, these case studies show that the near-term discriminator is not the channel dimensionality, but whether contacts/interfaces and variability can be controlled within BEOL integration limits [1, 4]. In the near term (0–5 years), LDMs are most likely to complement silicon technologies in a BEOL-compatible manner, where key targets include process compatibility (thermal budget/contamination control), variability metrics, and small-scale circuit demonstrations. In the mid term (5–10 years), LDMs are expected to be evaluated by wafer-scale manufacturability, including reproducible process windows, statistically controlled device distributions, and larger-scale array/circuit validation with reliability qualification. In the long term (> 10 years), LDMs may enable paradigm shifts such as monolithic 3D integration and/or new-physics devices, where system-level energy–performance benefits become the primary targets. Only by advancing all three paths synergistically can transistors thrive sustainably in the foreseeable future.

**Fig. 2:**
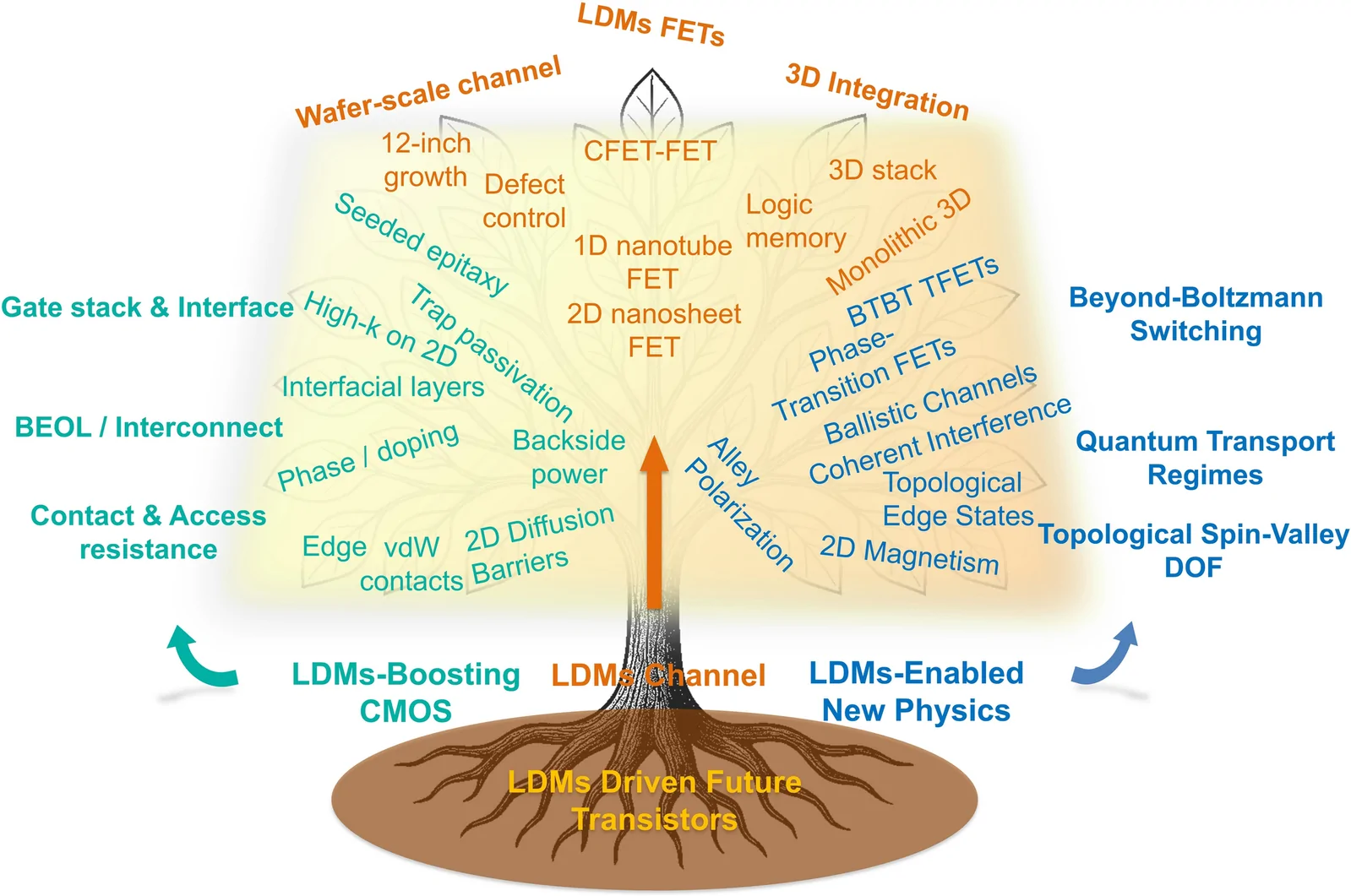
Roadmap of low-dimensional materials (LDMs) for future transistors across near (0–5 years), mid (5–10 years), and long term (> 10 years) horizons. The three pathways are: LDMs-boosting CMOS (BEOL-compatible integration), LDMs as channel materials (wafer-scale manufacturability and statistical control), and LDMs-enabled new physics (novel device concepts and longer-horizon integration) [1, 2, 4]

Notably, translating LDMs from lab to fab hinges on manufacturability and therefore requires coordinated advances from materials growth to device/integration modules and yield-relevant metrology. A primary bottleneck is wafer-scale, uniform and reproducible synthesis/integration of high-quality 1D/2D channels (e.g., wafer-scale CVD growth or transfer-free integration) [2]. Non-uniform thickness/grain/defect distributions translate into device-to-device variability (e.g., V_T and mobility spread), which in turn amplifies contact-resistance dispersion and degrades circuit yield [4]. In addition, transfer/alignment and interfacial contamination can introduce traps and uncontrolled barriers, making contacts and reliability difficult to qualify at scale [2]. Accordingly, actionable directions include wafer-scale process windows with statistically controlled distributions, contamination-aware interface/contact modules within BEOL thermal budgets, and standardized metrology/metrics that link device distributions to circuit yield and reliability [4]. Meanwhile, rapid progress in AI, brain–computer interfaces, flexible systems, and emerging computing is intensifying demand for heterogeneous integration and new device concepts, making the lab-to-fab transition both more urgent and more consequential.
